# QI Project Aiming to Reduce the Use of Restrictive Practice in Belfast Trust Psychiatry With the Implementation of a Therapy Cross

**DOI:** 10.1192/bjo.2024.396

**Published:** 2024-08-01

**Authors:** Sara McGucken, Jordan Taylor, Niall Corrigan, Pamela McGucken, Robert Dornan

**Affiliations:** Belfast Health and Social Care Trust, Belfast, United Kingdom

## Abstract

**Aims:**

The aims of the study were to illustrate the number and type of restrictive practices that were used across two inpatient wards within the Acute Mental Health Inpatient Centre in the Belfast trust over a two year period. This initially would highlight the prevalence and use of such practices and allow for comparison against the data collected after the implementation of the therapy cross. We hoped that with the implementation of the therapy cross we would see a decline in the use of physical interventions, use of IM medications and also the number of aggressive or distressing incidents and behaviours would also decrease.

**Methods:**

We utilized a statistical process control to collate and illustrate data. Daily data collection was carried out and compiled over a 2–3 year period and is ongoing with regards to ward incidents of aggressive behaviour, use of physical intervention, use of IM medications. In early October of 2023 the therapy cross was introduced and the run charts and data collection continued allowing for comparison of such behaviours and interventions pre and post intervention.

**Results:**

A percentage decrease of 50% of the weekly average was noted in incidents of aggressive and violent behaviours on one ward in AMHIC following implementation of therapy. A percentage decrease in average weekly use of IM injections was noted to be 13%. A 12% decrease was found in the use of physical intervention on a weekly average following the therapy cross.

**Conclusion:**

The implementation of a therapy cross in early October 2023 indicated improvement in the incidence of use of restrictive measures on two inpatient wards in the Belfast Trust, including the number of physical interventions such as holds that were required and also decreased the incidence of use of IM medications for rapid tranquillization. The data indicates a significant decrease in the number of cases of violent and aggressive behaviour on wards following implementation of a therapy cross.

